# Impact of oxLDL on Cholesterol-Rich Membrane Rafts

**DOI:** 10.1155/2011/730209

**Published:** 2011-01-18

**Authors:** Irena Levitan, Tzu-Pin Shentu

**Affiliations:** Section of Pulmonary, Critical Care and Sleep Medicine, College of Medicine, University of Illinois at Chicago, 840 South Wood Street, Chicago, IL 60612, USA

## Abstract

Numerous studies have demonstrated that cholesterol-rich membrane rafts play critical roles in multiple cellular functions. However, the impact of the lipoproteins on the structure, integrity and cholesterol composition of these domains is not well understood. This paper focuses on oxidized low-density lipoproteins (oxLDLs) that are strongly implicated in the development of the cardiovascular disease and whose impact on membrane cholesterol and on membrane rafts has been highly controversial. More specifically, we discuss three major criteria for the impact of oxLDL on membrane rafts: distribution of different membrane raft markers, changes in membrane cholesterol composition, and changes in lipid packing of different membrane domains. We also propose a model to reconcile the controversy regarding the relationship between oxLDL, membrane cholesterol, and the integrity of cholesterol-rich membrane domains.

## 1. Introduction

Oxidative modifications of LDL (oxLDL) are considered to be one of the major risk factors for the development of coronary artery disease (CAD) and plaque formation (reviewed in [1, 2]). Indeed, elevated levels of oxLDL are associated with an increased risk of CAD [3–5] and correlate with plasma hypercholesterolemia both in humans [6, 7] and in the animal models of atherosclerosis [8, 9]. It is also well-known that exposure to oxLDL induces an array of proinflammatory and proatherogenic effects but the mechanisms that underlie oxLDL-induced effects remain controversial. The prevailing hypothesis is that oxLDL results in loading cells with cholesterol inducing formation of cholesterol-laden macrophages (foam cells) and dysfunctional endothelial cells. However, growing number of studies have shown recently that the effects of oxLDL on membrane cholesterol homeostasis are complex and may actually involve cholesterol depletion and disruption of cholesterol-rich membrane domains (membrane rafts) rather than cholesterol loading. Membrane rafts were originally described as cholesterol- and sphingolipid-rich microdomains that provide platforms for protein-protein interactions in multiple signaling cascades [10–12]. A consensus definition for membrane rafts was suggested at the Keystone Symposium on Lipid Rafts and Cell Function (March 23–28, 2006 in Steamboat Springs, CO): “Membrane rafts are small (10–200 nm), heterogeneous, highly dynamic, sterol- and sphingolipid-enriched domains that compartmentalize cellular processes" [13]. Most recently, Simons and Gerl [11] further defined membrane rafts as “dynamic, nonoscale, sterol-sphingolipid-enriched, ordered assembles of proteins and lipids” that are regulated by specific lipid-lipid, protein-lipid, and protein-protein interactions [11]. The goal of this paper is to discuss the recent advances in our understanding of the impact of oxLDL on membrane rafts.

## 2. oxLDL: Definitions and Composition

The term oxidized LDL is used to describe LDL preparations which have been oxidatively modified *ex vivo* under defined conditions, or isolated from biological sources. The most typical procedure of LDL oxidation *ex vivo* is incubation of LDL with metal ions, Cu^2+^ in particular, that leads to the generation of multiple oxidized products in the LDL particle, including oxysterols, oxidized phospholipids, and modified apolipoprotein B (reviewed in [14, 15]). The oxidized LDL preparations described in the literature are broadly divided into two main categories: “minimally modified LDL” (MM-LDL) and (fully or extensively) oxidized LDL (oxLDL) based on the degree of LDL oxidation. Cu^2+^ oxidation of LDL can generate both minimally modified and fully oxidized LDLs depending on the duration of the exposure and ion concentration. Two other procedures that are also used to generate oxLDL *ex vivo* are enzymatic oxidation by 15-lypoxygenase or myeloperoxidase or by incubating LDL with 15-lypoxygenase expressing cells (e.g., [16–19]). It is important to note that while it is controversial whether Cu^2+^ oxidation occurs *in vivo*, it was shown that there are significant similarities between Cu^2+^ oxidized LDL and oxLDL found in atherosclerotic lesions [20]. Most of the studies that examined the impact of oxLDL on membrane rafts were performed using Cu^2+^-oxidized LDL.

## 3. oxLDL and Disruption of Caveolae

Caveolae are well known to be a morphologically distinct subpopulation of membrane rafts that contain multiple signaling complexes and play major roles in the regulation of cell signaling [26–28]. The first clue that oxLDL may disrupt caveolae structure came from a study of Smart et al. [29] showing that cholesterol oxidation results in translocation of caveolin from plasma membrane to Golgi. In this study, fibroblasts were exposed to cholesterol oxidase, an enzyme that converts cholesterol to cholestenone [30] and while cholestenone remained on the plasma membrane, this treatment resulted in the majority of caveolin (~60%) moving from the plasma membrane to Golgi [29]. Internalization of caveolin, in turn, was associated with a modest (10%) decrease in the number of caveolae [29]. Since it was shown in later studies that cells that are fully devoid of caveolin do not form caveolae [31, 32], a relatively minor effect induced by a partial loss of caveolin suggests that it is present in excess. Smart et al. suggested that the loss of caveolin due to oxidative modifications of cholesterol may be one of the mechanisms by which oxLDL disrupts endothelial function.

**Figure 1:**
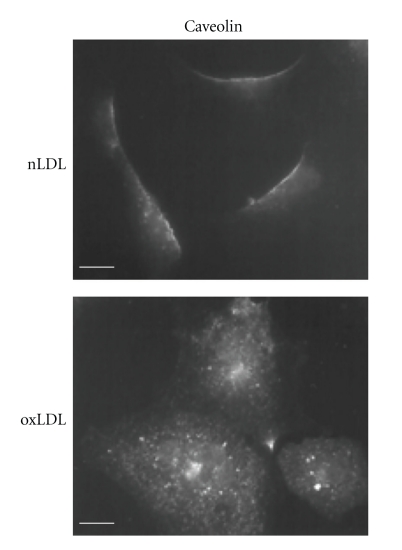
Oxidized LDL induces translocation of caveolin from the plasma membrane to intracellular compartments. Endothelial cells were treated with 10 *μ*g/mL of nonmodified LDL (nLDL) or oxLDL (15–20 nmol/mg TBARS) in 100% lipoprotein-defficient serum containing 20 mm HEPES (pH 7.4) for 1 h at 37°C. Caveolin was visualized with a rhodamine isothiocyanate-goat antirabbit IgG. Adapted from [21].

A more direct evidence for oxLDL-induced disruption of caveolae came from a later study of the same group [21] demonstrating that a short (30 min) exposure of porcine aortic endothelial cells to a relatively low dose (10 *μ*g/mL) of oxLDL results in internalization of caveolin (Figure 1) and a dramatic, virtually total decrease of cholesterol level in the caveolae fractions of these cells. Both the level and the degree of LDL oxidation (15–20 nmol/mg TBARS) used in this study were comparable with those found *in vivo* (7–35 *μ*g/mL, 11 nmol/mg protein TBAR) [3, 20]. Since both the structure and the function of caveolae critically depend on membrane cholesterol [33, 34], Blair et al. [21] concluded that oxLDL disrupts endothelial caveolae. Consistent with this conclusion, it was also shown that cholesterol depletion of caveolae resulted in internalization and inhibition of endothelial nitric oxide (NO) synthase (eNOS), a key enzyme, that is, regulated by caveolin and is responsible for endothelial NO synthesis [21]. Blair et al. [21] also showed that similar effects on eNOS were observed in response to cholesterol depletion supporting the notion that oxLDL-induced eNOS inhibition is related to cholesterol depletion. Consistent with these observations, a later study from the same group [35] showed that the level of caveolae cholesterol in oxLDL-treated endothelial cells can be preserved by exposing the cells to high-density lipoproteins (HDL), which in this case was suggested to serve as cholesterol donor and restore eNOS function. These observations led to a hypothesis that oxLDL may actually act as a cholesterol acceptor and remove cholesterol from the cellular membranes rather than load cells with cholesterol. It is not clear, however, how exactly oxLDL becomes a cholesterol acceptor. One theoretical possibility would be a direct exchange of lipids between the plasma membrane and the lipid core of the oxLDL particle. However, Uittenbogaard et al. [35] showed that oxLDL-induced depletion of endothelial caveolae and internalization of eNOS is mediated by the CD36 receptor, one of the major scavenger receptors that are responsible for the recognition and internalization of oxLDL, a pathway that leads to oxLDL degradation [36, 37], a detailed description of different oxLDL receptors is presented in our recent review and is beyond the scope of the current discussion [38]. An involvement of a scavenger receptor suggests that oxLDL needs to be internalized and possibly degraded to induce cholesterol depletion of caveolae. An alternative possibility would be that oxLDL binding to the CD36 receptors activates a signaling pathway that leads to cholesterol efflux. These mechanisms, however, are entirely not understood yet. Recently, we have shown that oxLDL results in fluidization of cholesterol-rich membrane domains and suggested that it might be attributed to a redistribution of cholesterol between cholesterol-rich and cholesterol-poor domains [25], a new paradigm for oxLDL-induced impact on membrane cholesterol. This possibility will be discussed in detail in the last part of this paper.

Another important clue about the mechanism that may underlie oxLDL-induced cholesterol depletion comes from the studies that focus on oxidized phospholipids. The first detectable products of LDL lipid oxidation are oxidized polyunsaturated fatty acids and the products of the oxidation, particularly oxidized products of palmitoyl-arachidonyl-phosphatidyl choline (oxPAPC), constitute one of the major active components of minimally oxidized LDL [39, 40]. Furthermore, oxPAPC were identified as the lipid moieties critical for the recognition of oxLDL by CD36 receptors [41]. Similar to oxLDL, oxPAPC also induced internalization of caveolin and depletion of cholesterol from caveolin-rich membrane fractions [39, 42]. These observations further underscored the role of CD36 receptors in the observed effects. 

Interestingly, the authors [43] have shown that diet-induced hypercholesterolemia in apoE-deficient mice (apoE^−/−^), a mouse model for atherosclerosis, also results in a dramatic decrease of the cholesterol level in caveolae isolated from whole blood vessels. Furthermore, since endothelial cells constitute only a single cell layer on the inner surface of the blood vessels while the majority of the vessel cells are contributed by smooth muscle cells, a decrease in the level of caveolae cholesterol in the whole vessel homogenate indicates that this effect is observed not only in endothelial cells but also in the smooth muscle cells. This study demonstrates that depletion of caveolae cholesterol may occur both *in vitro* and *in vivo*. More studies, however, are needed to evaluate the impact of oxLDL on caveolae on the structural level and to elucidate the mechanisms that underlie these effects.

## 4. oxLDL and Disruption of Noncaveolae Cholesterol-Rich Membrane Rafts

It is also well recognized today that cholesterol-rich membrane domains may form in the absence of caveolin indicating the existence of noncaveolae membrane rafts [28, 44]. The exact nature, morphology, size, density, and molecular composition of noncaveolae rafts in cellular membranes are still controversial, as summarized in multiple excellent reviews (e.g., [13, 45–48]) and a detailed discussion of these topics is beyond the scope of the current review. These noncaveolae membrane rafts are typically identified either by clustering of the proteins that are anchored to glycosylphosphatidylinositol (GPI-anchored proteins) [23, 49] or by ganglioside GM1, another major membrane raft marker [50]. Indeed, it was shown that while GPI-anchored proteins partition into detergent insoluble fractions that contain cholesterol-rich membrane domains, they are diffusely distributed at the cell surface and do not colocalize with caveolae except for after cross-linking [49, 51]. It is also important to note that while caveolae are estimated to constitute only about 2%–7% of the cell surface [34, 49], the surface area occupied by cholesterol-rich membrane domains appears to constitute a much larger area of the plasma membrane surface, as estimated by tracking various membrane raft markers. For example, tracing of single lipid molecules showed that about 15% of the plasma membrane is occupied by the regions of slow lipid motion that are expected to correspond to the ordered cholesterol-rich membrane raft domains [52]. Similar or even higher estimates (10%–40%) were obtained in other studies using different probes [25, 53–55]. It is generally believed, therefore, that noncaveolae rafts constitute a significant population of cholesterol-rich ordered membrane domains.

**Figure 2:**
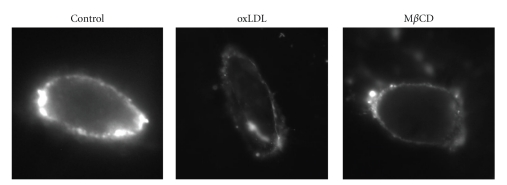
oxLDL indices internalization of GM1. Typical images of fluorescently labeled CTx staining in nonpermeabilized control, oxLDL-, and M*β*CD-treated bovine aortic endothelial cells showing the surface expression of G_M1_ on the plasma membrane. Similar to the earlier studies, cells were exposed to 10 *μ*g/mL oxLDL for 1 h at 37°C but the degree of LDL oxidation was a little lower (10–15 nmol/mg TBARS). For cholesterol depletion, cells were treated with 5 mM M*β*CD for 1 hour, a treatment that typically decreases cellular cholesterol level by 50%. Adapted from [22].

Several studies have shown that exposure to oxLDL results in significant redistribution of noncaveolae membrane raft markers. First, Zeng et al. [56] showed that exposure of Chinese Hamster Ovary cells (CHO) that overexpress CD36 receptors to oxLDL results in the internalization of GPI-anchored decay accelerating factor (DAF), one of the GPI-anchored proteins that was used earlier to analyze the differential distributions of GPI-anchored proteins and caveolin [49]. Surprisingly, even though caveolin is also expressed in CHO cells, in contrast to an earlier study in endothelial cells [21], it was not internalized in response to oxLDL. One possible explanation of this discrepancy is differential partitioning of CD36 receptors into different membrane domains in endothelial cells and in CHO cells. More recently, we have shown that exposure of endothelial cells to oxLDL also induces internalization of G_M1_ and that the effect of oxLDL was fully simulated by exposing the cells to methyl-*β*-cyclodextrin (M*β*CD), a cyclic oligosaccharide that binds to cholesterol with high affinity and depletes membrane cholesterol (Figure 2, [57]). Taken together, these studies suggest that oxLDL induces internalization of membrane raft protein complexes from the plasma membrane to the intracellular membranes. The similarity between the effects of oxLDL and cholesterol depletion on the distribution of G_M1_ between the plasma and the intracellular membranes supports the hypothesis that oxLDL disrupts cholesterol-rich membrane domains.

**Figure 3:**
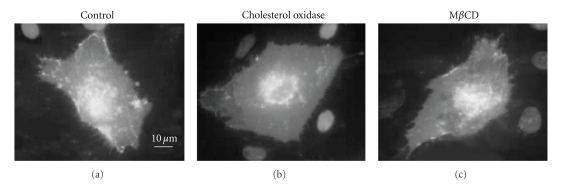
Visualizing noncaveolae lipid rafts by proximal imaging of GPI-anchored tt-GFP. Human umbilical vein endothelial cells are transfected with GPI-anchored temperature-tolerant green fluorescent protein (ttGFP) probe and exposed either to cholesterol oxidase (chol ox, 0.5 U/l) for 60 minutes or 2 mM methyl-*β*-cyclodextrin (M*β*CD) for 30 minutes. Adapted from [23].

Further insights into the impact of oxLDL on membrane rafts was recently obtained using a novel methodology called proximity imaging [23]. This approach is based on a property of a temperature-tolerant mutant of Green Fluorescent Protein (ttGFP) to undergo a shift in absorbance as a function of the distance of the neighboring molecules providing a unique tool to study molecule clustering [58]. Patschan et al. [23] used this approach to analyze the distribution of GPI-anchored ttGFP, as a probe to visualize membrane rafts in endothelial cells. As expected, exposing the cells to cholesterol oxidase or to M*β*CD resulted in significant decrease of the ttGFP clusters on the membrane (Figure 3). Furthermore, the same effect was also observed in cells exposed to cholesterol oxidase. It is a little surprising, though, that in this study GPI-anchored ttGFP fluorescence occupied only less than 2% of the cell surface, a much lower estimate that was obtained by other methods. An intriguing possibility is that GPI-anchored proteins represent a small specific subpopulation of membrane rafts. Alternatively, this discrepancy may also be due to the efficiency of GPI-ttGFP overexpression. In this study, oxLDL by itself did not have a significant effect on the surface area of the rafts, as estimated by clustering of the GPI-ttGFP but exposing the cells to oxLDL in the presence of dimethyl-L-arginine dihydrochloride (ADMA), an inhibitor of NO synthesis, resulted in a significant effect even though ADMA by itself also had no effect [23]. These observations suggested that oxLDL disrupts membrane rafts in a NO-dependent way but the exact mechanism underlying these effects is not clear. Patschan et al. [23] proposed that oxLDL-induced modifications of the lipid composition of the membrane play a key role in the disruption of GPI-ttGFP clustering. Thus, multiple studies demonstrate that oxLDL may induce disruption of membrane rafts, as estimated by the internalization or unclustering of different membrane raft markers.

## 5. oxLDL and Formation of Ceramide Platforms

Several studies have shown that oxLDL also induces hydrolysis of sphingomyelin (SM) (e.g., [59–63]), a second major lipid component of membrane rafts (reviewed in [13, 64]). SM hydrolysis is expected to disrupt the integrity of membrane rafts, alter cholesterol distribution between the cellular membranes, and increase the level of membrane ceramide, which by itself may form tightly packed ceramide-rich microdomains (reviewed in [48, 64]). Indeed, multiple studies have shown that oxLDL-induced hydrolysis of sphingomyelin is accompanied with a concomitant increase in the level of cellular ceramide [59–63]. Furthermore, Grandl et al. [65] have recently shown that oxLDL results in the formation of large ceramide-rich membrane rafts in human macrophages that could be detected by fluorescent microscopy. It is also important to note that hydrolysis of sphingomyelin and an increase in the cellular ceramide levels were implicated in several of oxLDL/oxPC-dependent cellular processes, such as oxLDL-induced proliferation of vascular smooth muscle cells [59] and apoptosis of vascular endothelial cells [62] and macrophages [60] and oxPC-induced synthesis of an inflammatory cytokine interleukin-8 [66]. Specific pathways, however, involved in the diverse ceramide-induced cellular effects have been summarized in a number of excellent reviews and are beyond the scope of this review (e.g., [48, 67, 68]). An interesting question is whether oxLDL-induced disruption of cholesterol-rich membrane domains and formation of ceramide-rich domains are mutually dependent. Indeed, since SM has high affinity for cholesterol, SM hydrolysis may be expected to result in a partial loss of cholesterol from SM-rich membrane domains. Consistent with this idea, SM hydrolysis induced by exposing cells to a bacterial enzyme Sphingomyelinase D was shown to induce an increase in cholesterol internalization and esterification both in macrophages and in endothelial cells [25, 69]. Since it is also known that cholesterol esterification requires its internalization from the plasma membrane to the intracellular compartments, we tested a possibility that oxLDL may induce cholesterol depletion of the plasma membrane cholesterol-rich domains by inducing its internalization and esterification. Our observations, however, did not support this possibility because at least in endothelial cells we detected no measurable increase in cholesterol esterification in response to oxLDL [25]. Also, as described below, while we found strong similarities between the effects of oxLDL and cholesterol depletion on endothelial biomechanical properties, effects of SM hydrolysis were entirely different [25]. More studies, however, are needed to elucidate possible links between oxLDL, cholesterol, and SM in different cell types.

## 6. oxLDL and Cholesterol Depletion

In spite of the mounting evidence that oxLDL may disrupt the integrity of cholesterol-rich membrane rafts, as judged by the redistribution of different raft markers, direct evidence for the ability of oxLDL to actually remove cholesterol from the cellular membrane remains elusive and controversial. Specifically, while some studies have demonstrated oxLDL/oxPC-induced cholesterol depletion, mostly from the caveolae fraction of the plasma membrane [21, 42, 43], other studies showed no significant effect [22, 25, 70] neither in cholesterol-rich nor in cholesterol-poor membrane fractions [22]. Most recently, we showed that oxLDL indeed slightly facilitates cholesterol efflux from endothelial cells but the general effect is very mild and has no significant impact on the total level of cholesterol in the membrane [25]. Also, as described above, we found no evidence that oxLDL induces internalization of cholesterol to the intracellular membranes, as assayed by cholesterol esterification [25]. However, while direct evidence for oxLDL-induced cholesterol depletion is very limited, a growing number of studies demonstrate striking similarities between the effects of oxLDL and of cholesterol depletion on an array of different cellular functions, providing indirect indication that the impact of oxLDL on cellular functions is related to cholesterol depletion.

**Figure 4:**
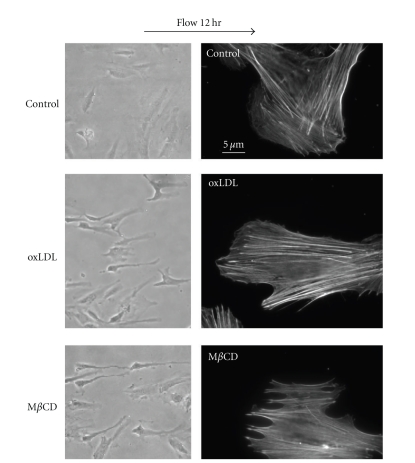
oxLDL and cholesterol depletion have similar effects on realignment of endothelial cells in the direction of the flow. *Left column:* typical images of control, oxLDL-treated cells (10 *μ*g/mL oxLDL, 1 h), and M*β*CD-treated cells (5 mM, 1 h) exposed to 10 dyn/cm^2^ for 12 hours. *Right column:* typical images of F-actin structure in the same cell populations. Arrow indicates the direction of flow. Adapted from [24].

As described above, earlier studies by Blair et al. [21] showed that oxLDL-induced inhibition of endothelial nitric synthase (eNOS) can be simulated by M*β*CD-induced cholesterol depletion and abrogated by maintaining cellular cholesterol at a constant level [35]. Similarly, oxPAPC-induced production of an inflammatory cytokine Interleukin-8 was also simulated by M*β*CD-cholesterol depletion and prevented by cholesterol loading [42]. Furthermore, Yeh et al. [42] showed that oxPAPC also results in a sustained activation of sterol regulatory element-binding protein (SREBP) and induction of SRBEP-targeted genes (LDLR and HMG CoA synthase). Since it is well known that SREBPs are regulated by the level of cellular cholesterol and activated by cholesterol depletion [71], the ability of oxPAPC to activate SREBP is consistent with the observation that oxPAPC induces cholesterol depletion. More recently, our studies have shown that multiple effects of oxLDL on endothelial biomechanical properties can also be simulated by cholesterol depletion. First, we have shown that exposure to oxLDL and depletion of membrane cholesterol result in an increase in endothelial stiffness, as estimated by measuring progressive membrane deformation in response to negative pressure [22, 72] or by atomic force microscopy [25]. The same correlation was observed also for the ability of the cells to generate force on the cell-substrate interface [22, 25], to form endothelial networks in 3D collagen gels [22, 25] and to realign in the direction of the flow ([24], Figure 4). Furthermore, the similarities between the effects of oxLDL and M*β*CD on endothelial realignment in the direction of the flow are apparent both on the level of single cells and of individual F-actin fibers ([24], Figure 4). A correlation between the effects of oxLDL and cholesterol depletion across an array of different cellular functions suggests that there is a common mechanistic denominator. However, it is also possible that the common denominator is not cholesterol depletion but rather a downstream step that can be activated by both oxLDL and by cholesterol depletion independently. To address this possibility, we tested whether the effects of oxLDL can be reversed by increasing the level of membrane cholesterol. Indeed, our further studies have shown that all of the effects of oxLDL on endothelial biomechanical properties could be reversed by supplying the cells with a surplus of cholesterol either by sequential exposing the cells to acLDL [24] or by sequentially exposure to oxLDL and then to M*β*CD-cholesterol [25]. These observations indicate that oxLDL-induced effects on endothelial biomechanical properties are cholesterol dependent. It is important to note that enriching endothelial cells with cholesterol in the absence of oxLDL had no effect on endothelial biomechanics [25, 72] indicating that the reversibility is not a result of simple cancellation of the two opposite effects. Thus, multiple studies show a remarkable similarity between the effects of oxLDL and of M*β*CD on endothelial properties. In contrast, we found no similarities between the effects on oxLDL and SM hydrolysis on endothelial biomechanics suggesting that these effects cannot be attributed to oxLDL-induced formation of ceramide platforms [25]. 

Most surprisingly, no changes in cellular cholesterol were observed in the same studies that showed that oxLDL-induced effects are simulated by cholesterol depletion and reversed by cholesterol enrichment [22, 25]. To resolve this discrepancy, we proposed a hypothesis that oxLDL may alter the lateral distribution of membrane cholesterol, which in turn may disrupt cholesterol-rich membrane domains and induce cholesterol depletion-like physiological effects. This hypothesis was addressed in our recent study by estimating lipid packing of cholesterol-rich and cholesterol-poor membrane domains in cells exposed to oxLDL.

## 7. Impact of oxLDL on Lipid Packing of Membrane Domains in Living Cells

Numerous studies have examined physical properties of cell membranes under different experimental conditions using probes that are sensitive to membrane fluidity/lipid packing. However, until recently these approaches did not allow examining the heterogeneity of the biophysical properties of the biological membranes in living cells. A major breakthrough in alleviating this constraint was developing Laurdan two-photon imaging, a novel approach to probe lipid order of different membrane domains in living cells [53, 73]. The general principle of this technique is that Laurdan dye is sensitive to the polarity of the local environment and undergoes a red shift as the phase boundary of the lipid bilayer changes from gel to fluid [53, 74]. When using this approach, cells typically present a punctuate distribution of membrane domains that range in their biophysical properties from fluid to ordered, as estimated by general polarization (GP) ratio that reflects lipid packing: the higher the GP value, the more ordered is the domain [53, 73]. Furthermore, the distribution of membrane domains between fluid and ordered can be analyzed quantitatively by analyzing the distributions of the GP values for individual cells. More specifically, the histograms of the GP values are typically fitted with a two-Gaussian distribution with the two peaks representing ordered (peak with higher GP values) and disordered (lower GP values) membrane domains [53, 73]. 

Earlier studies have shown that, as expected, cholesterol depletion results in a significant shift to less ordered membrane structure, as indicated by a decrease in the GP values corresponding to a decrease lipid packing [53, 74]. It is also important to note that M*β*CD-induced cholesterol depletion results in a decrease in lipid packing/fluidization of both ordered and disordered domains, as apparent from a decrease in the GP values for both types of the domains. Surprisingly, the areas that are covered by the ordered and fluid domains do not change significantly in cholesterol-depleted cells suggesting that M*β*CD affects the physical properties of these domains rather than their coverage of the cell surface. Our recent observations are fully consistent with these studies [25]. These observations also provided further insights into the relationship between cholesterol and different membrane domains. Indeed, one of the common misconceptions in the membrane raft field is an assumption that M*β*CD removes cholesterol specifically from the raft domains and therefore interpreting the effects of cholesterol depletion as direct evidence for the involvement of membrane rafts. In contrast, several studies have shown that careful examination of the lipid composition of cholesterol-rich and cholesterol-poor membrane domains demonstrates that M*β*CD removes cholesterol from both types of the domains (reviewed in [75]). This conclusion is further confirmed by Laurdan imaging demonstrating that, as described above, both types of the domains become more fluid. Conversely, we have shown that cholesterol enrichment increases the GP values in both ordered and fluid membrane domains suggesting that, as expected, the cholesterol enrichment tightens lipid packing in both types of the domains [25].

**Figure 5:**
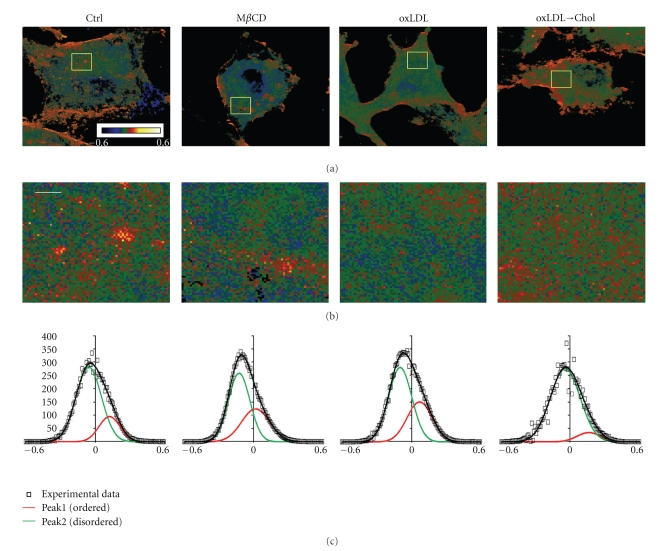
Impact of oxidized low-density lipoprotein (oxLDL) on lipid packing of membrane domains in bovine aortic endothelial cells. (a) Typical general polarization (GP) images of control cells (Ctrl), M*β*CD-treated cells, oxLDL-treated cells (oxLDL), or cells exposed to oxLDL and M*β*CD-cholesterol sequentially (oxLDLChol). Scale bar is 5.6 *μ*m. (b) zoom-in representative regions of the GP images shown in (a) (the zoomed regions are 5.6 *μ*m × 5.6 *μ*m). Scale bar is 1 *μ*m. (c) GP histograms for the four experimental cell populations (dots) fitted by a two-Gaussian distribution with the curve representing ordered domains (red) and the curve representing fluid domains (green). The sum of the Gaussians is shown in black. Adapted from [25].

Most importantly, employing Laurdan two-photon imaging allowed us to obtain new insights into the impact of oxLDL on the membrane structure of endothelial cells [25]. Specifically, we have shown that, consistent with the idea that oxLDL induces disruption and cholesterol depletion of cholesterol-rich membrane domains, exposure to oxLDL indeed resulted in a shift to less ordered membrane structure, particularly of the ordered domains (Figure 5). Conversely, exposure of oxLDL-treated cells to M*β*CD-cholesterol resulted in a partial reversal of its effect on the properties of the ordered domains. However, while there is again significant similarity between the effects of oxLDL and cholesterol depletion, the two are not identical. The main difference between oxLDL and M*β*CD is that oxLDL affects primarily the ordered domains with no significant effect on the fluid domains whereas M*β*CD-induced cholesterol depletion affects both. In addition, while cholesterol depletion also results in the overall decrease of the general GP values that reflect the packing of the entire membrane, oxLDL has no effect. A decrease in general GP values in cholesterol-depleted cells is not surprising and is fully consistent with the overall decrease in membrane cholesterol. The second observation is more significant suggesting that the total amount of cholesterol in the membrane does not change significantly. This indeed is consistent with the lack of oxLDL effect on the level of membrane cholesterol as estimated by other methods. We proposed, therefore, that oxLDL may disrupt membrane rafts not by removing membrane cholesterol but rather by incorporation of oxysterols into the membrane.

## 8. Impact of Oxysterols on Membrane Rafts

Oxysterols are found in abundance in Cu^2+^-oxidized LDL, in which cholesterol is oxidized preferably at 7 positions resulting in the generation of 7-ketocholesterol, 7*β*-hydroxycholesterol, and 7*α*-hydroxycholesterol [76]. In addition, 27-hydroxycholesterol, that is, generated *in vivo,* has been shown to accumulate in foam cells in atherosclerotic lesions [76]. Several studies have shown that oxysterols result in inhibition of cholesterol efflux in the mouse and it was suggested that impairment of cholesterol homeostasis by the inhibition of cholesterol efflux may be mechanism by which oxysterols affect cellular function [77, 78]. Interestingly, 7-ketocholesterol was shown to deplete cholesterol specifically from the raft domains in human macrophages [79] and disrupt lipid packing of the immunological synapses in sterol-enriched T lymphocytes [80] and in cholesterol-rich membrane domains in endothelial cells [25]. These observations suggest that incorporation of oxysterols may also play an important role in oxLDL-induced disruption of cholesterol-rich membrane domains.

## 9. Conclusions

**Figure 6:**
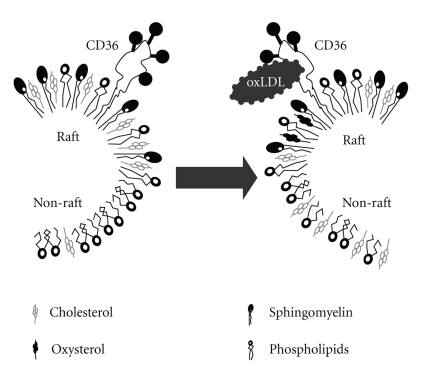
A model proposed for the impact of oxLDL on the lateral distribution of cholesterol between raft and nonraft domains.

So, how can we reconcile the following observations: (i) exposure to oxLDL results in internalization/dispersion of membrane raft markers but has only small or no effect on the level of membrane cholesterol; (ii) multiple effects of oxLDL on cellular function can be simulated by cholesterol depletion or direct exposure to 7-ketocholesterol and reversed by cholesterol enrichment; (iii) exposure to oxLDL induces a decrease in lipid packing, specifically in cholesterol-rich ordered membrane domains? To reconcile these observations, we propose a new model for the impact of oxLDL on membrane rafts (Figure 6). First of all, we propose that oxLDL induces a lateral redistribution of cholesterol between ordered domains and fluid domains, which results in a decrease in lipid packing of the ordered domains but no significant change in the more abundant fluid domains. We also propose that direct insertion of oxysterols into the plasma membrane may contribute to this effect. Clearly, more studies are required to test this paradigm. 

While elevated levels of oxLDL are associated with hypercholesterolemia and development of atherosclerosis, the propensity of the evidence suggests that oxLDL results in disruption of cholesterol-rich membrane domains and either depletion or lateral re-distribution of cholesterol between the ordered and the fluid domains of the membrane. Furthermore, multiple oxLDL-induced changes in cellular functions, particularly in endothelial cells, are apparently related to the disruption of cholesterol-rich domains rather than to cholesterol enrichment. These conclusions may have a major impact on our understanding of the mechanisms of oxLDL function and the role of membrane rafts in oxLDL-induced pathological effects.
